# CircRNA: A new class of targets for gastric cancer drug resistance therapy

**DOI:** 10.3389/pore.2023.1611033

**Published:** 2023-03-30

**Authors:** Ying Zheng, Zhe Li, Yao Wang, Wanjiao Chen, Yifan Lin, Junming Guo, Guoliang Ye

**Affiliations:** ^1^ Department of Biochemistry and Molecular Biology, Zhejiang Key Laboratory of Pathophysiology, Medical School of Ningbo University, Ningbo, China; ^2^ Department of Gastroenterology, The First Affiliated Hospital of Ningbo University, Ningbo, China; ^3^ Institute of Digestive Diseases of Ningbo University, Ningbo, China

**Keywords:** gastric cancer, drug resistance, circular RNA, therapeutic target, biogenesis

## Abstract

Gastric cancer (GC) is one of the most common malignancies worldwide. Patients with advanced GC need palliative care to ensure survival. This includes the use of chemotherapy agents, such as cisplatin, 5-fluorouracil, oxaliplatin, paclitaxel, and pemetrexed, as well as targeted agents. However, the emergence of drug resistance evidence in poor patient outcomes and poor prognosis is a motivation to determine the specific mechanism of drug resistance. Interestingly, circular RNAs (circRNAs) play an important part in the carcinogenesis and progression of GC and are involved in GC drug resistance. This review systematically summarizes the functions and mechanisms of circRNAs underlying GC drug resistance, especially chemoresistance. It also emphasizes that circRNAs can serve as promising targets for improving drug resistance and therapeutic efficacy.

## Introduction

Cancer is one of the primary causes of death. Gastric cancer (GC) is a type of gastrointestinal tumor with an incidence that ranks it among the top five cancers and mortality rates that place it among the top four (1). The main reason for the high GC mortality rate is likely due to its diagnosis in the advanced or metastatic stage (2). At that time, radical surgery is no longer a feasible option and palliative therapy is necessary to improve the survival rate of patients (3). Chemotherapy, targeted therapy, and immunotherapy are novel methods that can be used as an adjunct combined with surgery or as the main treatment for advanced cancer (4). For example, research in gastroesophageal cancer patients who have underwent postoperative adjuvant chemotherapy has shown that the overall survival at 1, 3, and 5 years in the adjuvant chemotherapy group was 5% higher than that in the resection alone group (5). However, one small study has shown that when patients with advanced GC were divided into three groups based on their sensitivity to chemotherapy drugs, the one-year survival rate in the sensitive group was significantly higher than that in the resistant group, whereas the time to progression in the sensitive group was significantly longer than that in the resistant group (6). Based on this, chemoresistance has become a barrier to existing treatments, which has a significant impact on the follow-up treatment effect and patient survival rates. In another trial, researchers have classified advanced GC after clinical sequencing based on biomarkers (7). Among them, the GC therapeutic drug capivasertib is a selective pan-AKT serine/threonine kinase (*AKT*) inhibitor with the ability to inhibit kinase activity of three *AKT* subtypes. The sequencing results have shown that GC can be divided into microsatellite instability-high (MSI-H) GC, microsatellite-stable (MSS) tumor, genomically stable, or mesenchymal subtype groups based on phosphatidylinositol-4,5-bisphosphate 3-kinase catalytic subunit alpha (*PIK3CA*) hot spot mutations (E542K, E545K, and H1047R) (7). Researchers have observed that different GC subtypes have different sensitivity to the capivasertib treatment. Among the eight patients with E542K mutation, four had a persistent response to the combination of capitaserib/paclitaxel (PTX), and none of the four patients with H1047R *PIK3CA* mutations responded to capivasertib. Thus, identifying specific targets can improve GC sensitivity to specific drugs (7). In general, a variety of new treatment modalities have been used alone or as a supplement in addition to surgery for cancer treatment. It is very important to improve and refine the above methods, including reducing the rate of chemotherapy resistance.

### Circular RNAs have potential as drug resistance targets

**FIGURE 1:**
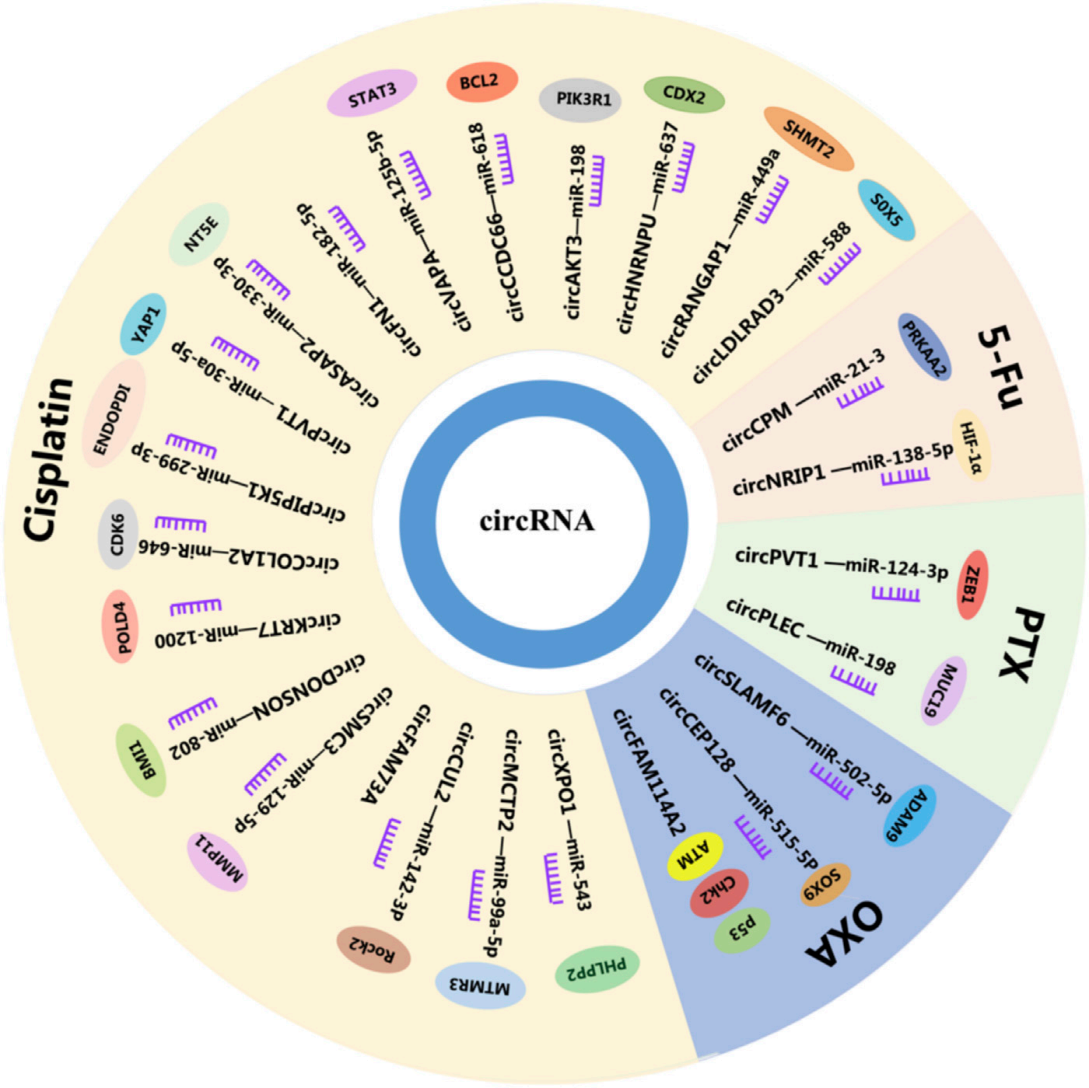
CircRNAs in GC. CircRNAs play a vital role in drug resistance by interacting with miRNAs and mRNAs, circRNAs, circular RNAs; miRNAs, microRNAs; mRNAs, and messenger RNAs.

Circular RNAs (circRNAs) are formed by alternative splicing of the 5′ and 3′ ends of RNA molecules. They are significantly more stable than the associated linear RNAs and demonstrate resistance to RNase R digestion (8). Therefore, circRNAs have a unique covalently closed circular structure that gives them stability and the potential as a target for a variety of therapies. For example, circAKT3 is significantly overexpressed in cisplatin (CDDP)-resistant GC tissues and cells and eliminates the inhibition of miR-198 to activate the *PI3K/AKT* signaling pathway in GC cells, leading to CDDP-based DNA-damaging chemotherapy resistance (9) (Figure 1; Supplementary Table S1). However, the molecular mechanisms by which circRNAs regulate the therapeutic effects of drugs in GC remain unclear. Further research is needed to study the drug resistance mediated by circRNAs in GC. The present review summarizes the roles of circRNAs in drug resistance during several GC treatments and discusses their potential mechanisms. Clinical application of circRNAs as a therapeutic target needs to be discussed further.

### Biogenesis and functions of circular RNAs

**FIGURE 2:**
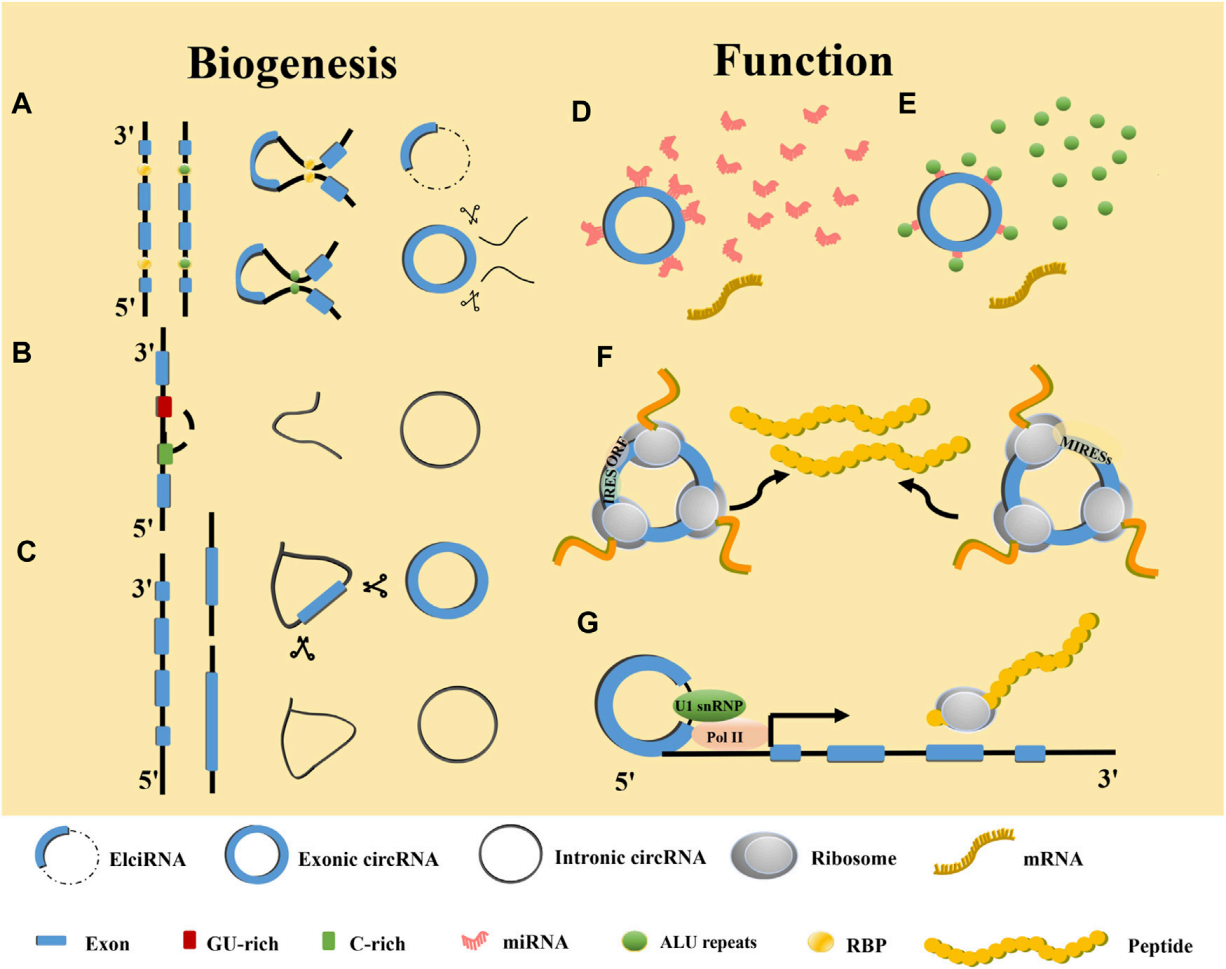
Biogenesis and functions of circRNAs. **(A)** RBP and ALU-mediated circRNA generation. **(B)** Mechanism of intronic circRNA generation. **(C)** Lariat-driven mediated circRNA generation. **(D)** Function as miRNA sponge. **(E)** Interaction with RBPs. **(F)** Translation into proteins or polypeptides. **(G)** Enhancer of gene expression.

CircRNAs are stable covalently closed ncRNAs that were first identified in plant viruses in the 1970s (10). They were deemed to be miscleavage byproducts during precursor messenger RNA (pre-mRNA) processing (11). With the development of biomolecular techniques, circRNAs have been found to have potential biological functions that play vital roles in cancers (8). In addition, circRNAs can be divided into exon-derived circRNAs (ecRNAs) (8), exon-intron circRNAs (EIciRNAs) (12), and intron-derived circRNAs (ciRNAs) based on their composition (13). The ecRNAs can be formed *via* intron- or lariat-driven circularization (Figures 2A, C). The mechanism for intron-driven circularization is mediated by base pairing between introns located on the exon flanks (Alu repeats or non-repeating elements with a length of about 300 nt) or dimerization of RNA-binding proteins (RBPs), which leads to the tight binding of the upstream 3′ splice site to the 5′ splice site that forms a circular structure after the introns are removed. The mechanism for lariat-driven circularization relies on exon skipping in pre-mRNA during post-transcription and can decrease the distance between non-adjacent exons. Furthermore, ecRNAs can be formed by removing introns. EIciRNAs are formed by intron-driven circularization (Figure 2B). They are generated when the introns between the acceptor and the donor are not removed. In addition, ciRNAs that originate from tRNA intronic circular RNAs or pre-mRNAs are produced by forming a 2ʹ-5ʹend linkage. This linkage is a branchpoint between the 5ʹ splice site after the release of the 3ʹ exon and terminal 2ʹ-OH intron group (Figures 2B, C) (14).

With the advent of the sequencing technology age, more and more circRNA have been discovered to be widely expressed in eukaryotes and participating in various human pathological and physiological processes (15). The circRNAs affect numerous cancer hallmarks by sponging microRNA (miRNA), mutual effecting with and translating into proteins, regulating gene transcription, and competing with mRNA linear splicing. Among them, miRNA sponging is the most iconic circRNA feature (Figure 2D). For example, circVAPA is upregulated in CDDP-resistant GC cell lines (16). It has been reported that constitutive activation of signal transducer and activator of transcription 3 (STAT3) contributes to the chemotherapy resistance of GC cells (16). Researchers have found that circVAPA as a sponge for miR-125b-5p can act on STAT3 in GC *via* the miR-125b-5p/STAT3 axis to promote chemotherapy resistance of GC (17) (Figure 1; Supplementary Table S1). RBPs regulate at the post-RNA transcriptional level (Figure 2E) (18). For instance, heterogeneous nuclear ribonucleoprotein U-like 1 (HNRNPUL1) belongs to the family of RBPs and is involved in DNA damage repair. Through regulation of CDDP sensitivity-inhibited circMAN1A2 formation, HNRNPUL1 can inhibit CDDP sensitivity of esophageal cancer (19). In addition to interacting with RBPs, circRNAs also function as protein scaffolds, decoys, and recruiters (16, 20–22). The translation of circRNAs requires an internal ribosome entry site sequence (IRES) located in RNA to initiate cap-independent translation (23). IRES initiates the open reading frame for translation (24). In addition, m6A-induced ribosomal binding sites may be another mechanism that is independent of cap translation (Figure 2F) (25). ASK1-272a.a is a novel protein encoded by circASK1 that mediates the chemosensitivity-inducing effect of epidermal growth factor receptor (*EGFR*)-mutant lung adenocarcinoma (26). Furthermore, ASK1-272a.a has a function of competing with ASK1 for Akt1 binding, which competes with Akt1-induced ASK1 phosphorylation and inactivation, resulting in the activation of ASK1-induced apoptosis and alleviating gefitinib resistance (26). EIciRNAs and intron-derived RNAs are located in the nucleus, where they perform transcriptional regulation and other functions (27, 28). Studies have shown that EIciRNAs form the EIciRNA-U1 snRNP complex by binding with U1 snRNP, which ultimately enhances gene expression after interacting with Pol II at the gene promoter (28). Similarly, intron-derived RNAs can adjust the transcription of their parental genes by facilitating the elongation activity of Pol II (Figure 2G) (13). Flanking intron sequences are the main factor determining the efficiency of exon circularization. Moreover, circRNAs containing cyclization-promoting sequences can result in reduced efficiency of the flanking exon linear splicing, thereby competing with linear splicing of mRNAs (29).

In conclusion, circRNAs play important roles in cancer through their various functions. Multiple pathways dominated by circRNAs regulate the expression of the downstream genes. Therefore, an in-depth study of specific circRNA mechanisms in cancer drug resistance is crucial, which will contribute to the rapid development of cancer treatment.

## The circular RNAs and gastric cancer drug resistance

### The circular RNAs and chemoresistance

Chemotherapy has shown good efficacy as the main treatment for advanced cancer, but the occurrence of chemotherapy resistance hinders the integrity of the course of treatment and is a mainspring for chemotherapy failure in improving patients (30). Diverse mechanisms are involved in drug resistance, including DNA damage repair, operation of cell death-related pathways, regulation of cell viability and proliferation, modification of drug efflux, and metabolism (31), in which circRNAs play a crucial part. Moreover, circFAM114A2 is downregulated in GC and sponged miR-421 to upregulate the expression level of *ATM* (ATM serine). At the same time, circFAM114A2 inhibits the chemoresistance of L-OPH (Oxaliplatin) through the activation of the *ATM/Chk2* (checkpoint kinase 2)/*p53*-dependent pathway. *ATM* is a master gene regulator in the DNA damage response. Its activation leads to the execution of DNA repair or initiation of apoptosis (32). Furthermore, circHECTD1 expression is significantly upregulated in GC and facilitates GC cell glutaminolysis, growth, and invasiveness (33). In addition, circHECTD1 has been found to target miR-1256 to regulate ubiquitin-specific peptidase 5 expression levels and modulate the downstream β-catenin/*c-Myc* signaling pathway (33). Therefore, circHECTD may serve as a latent chemotherapy drug target for GC treatment (Figure 1; Supplementary Table S1).

### Cisplatin resistance of circRNAs in gastric cancer

CDDP is the first-line chemotherapy drug for GC that blocks transcription and DNA replication by forming intra-strand DNA cross-links, resulting in apoptosis (34). However, tumor cells can develop resistance to CDDP by interfering with CDDP-induced apoptosis *via* unique mechanisms, which ultimately limits the overall medical efficacy of the treatment (35).

The mechanisms of resistance to CDDP are varied, in which circRNAs play an important part. In particular, circCUL2 inhibits the proliferation, migration, invasion, and drug resistance of GC cells (36). Studies have shown that abnormally activated autophagy induced by chemotherapeutic drugs can offer energy to cancer cells (36). Interestingly, circCUL2 inhibits autophagy in CDDP-resistant GC cells through miR-142-3p/*ROCK2* (Rho-associated coiled-coil containing protein kinase 2), thus affecting the CDDP sensitivity of GC cells (36). Researchers have found that circCCDC66 was significantly overexpressed in CDDP-resistant GC cells (37). In addition, circCCDC66 induces CDDP resistance in GC cells *via* miR-618, significantly inhibiting the mRNA and protein levels of *BCL2* (BCL2 apoptosis regulator), which is a key regulator of the apoptosis signaling pathway (37). Sun et al. have found that circMCTP2 upregulation suppressed the proliferation of CDDP-resistant GC cells and induced CDDP-resistant GC cell apoptosis (38). It also reduced autophagy in CDDP-resistant GC, which has been demonstrated to promote resistance to tumor chemotherapy. In addition, as a sponge of miR-99a-5p, circMCTP2 inhibits the resistance of GC to CDDP by upregulating myotubularin-related protein 3 (*MTMR3*). Therefore, circMCTP2 might be a new therapeutic strategy for interference with CDDP resistance in GC (38). Furthermore, circFN1 is a novel circRNA that reinforces CDDP resistance in GC by sponging miR-182-5p, which has the capacity of activating apoptosis *via* the caspase-3 signaling pathway (39). Moreover, circFN1 functions as a ceRNA, abolishing the ability of miRNA to activate apoptosis and enhancing CDDP resistance (39). In addition, circASAP2 is upregulated in CDDP-resistant GC tissues and cells. Knockdown of circASAP2 promotes CDDP sensitivity and inhibited malignant behaviors of CDDP-resistant GC cells by targeting the miR-330-3p/*NT5E* (5′-nucleotidase ecto) axis (40). Zhang et al. have found that enhancing the expression of circXPO1 promoted the apoptosis of CDDP-resistant GC cells and inhibited their growth and metastasis. In other words, circXPO1 has the ability to sensitize the GC patients to CDDP therapy (41). Knockdown of miR-543 can promote tumor-suppressor factor leucine-rich repeat protein phosphatase 2 (*PHLPP2*), which can impact CDDP-resistant cell behaviors and chemotherapy sensitivity. Thus, circXPO1 can act as a sponge for miR-543 and be a latent therapeutic agent against CDDP-resistant GC (41). Prior research has shown that circDONSON can promote the resistance of GC cells to CDDP (42). In addition, miR-802 was determined to be a tumor suppressor in many human cancers. Targeting circDONSON can promote CDDP resistance in GC cells. Furthermore, BMI1 proto-oncogene (*BMI1*) is an oncogene that was found to be an miR-802 target that was overexpressed in CDDP-resistant GC tissues and cells (42). Therefore, these findings demonstrated that circDONSON regulated the sensitivity of CDDP resistance in GC *via* the miR-802/*BMI1* axis (42). Exosomes are endocytosis extracellular lipid bilayer vesicles that can transport specific proteins or nucleic acids to the corresponding recipient cells through intercellular communication mechanisms (43, 44). They are stable in a variety of extracellular fluids, including blood and urine (45). It is important that circRNAs are enriched and stable in exosomes, and that exosomal circRNAs can regulate the occurrence and development of GC by acting as a sponge of miRNAs (46). Exosomal circPVT1 might be a prospective indicator for GC in CDDP therapy (47). CircPVT1 knockdown suppressed CDDP resistance in CDDP-resistant GC cells by promoting apoptosis and inhibiting invasion or autophagy by negatively targeting miR-30a-5p. Yes1-associated transcriptional regulator (*YAP1*) is a direct target of miR-30a-5p, and reduction of its level by miR-30a-5p can promote CDDP resistance (47). In addition, circPIP5K1A knockdown enhances CDDP sensitivity of GC *via* the miR-299-3p/*ENDOPDI* (thioredoxin domain containing 5) axis (48). CircCOL1A2 induces GC CDDP resistance by effecting the miR-646/*CDK6* (cyclin dependent kinase 6) pathway (49). CircKRT7 heightens CDDP resistance in GC *via* the miR-1200/P*OLD4* (DNA polymerase delta 4) pathway (50). CircSMC is an miR-129-5p sponge that can promote the CDDP resistance of GC by upregulating matrix metallopeptidase 11 (*MMP11*) (51). In addition, circFAM73A has the ability to facilitate *in vitro* CDDP resistance in GC (52). CircHNRNPU facilitates cisplatin resistant of GC through miR-637/caudal type homeobox 2 (*CDX2*) (53). Exosomal circRANGAP1 promotes GC resistance to CDDP by regulating the miR-449a/serine hydroxymethyltransferase 2(*SHMT2*) axis (54). circLDLRAD3 knockdown can reduce CDDP resistance by regulating miR-588/SRY-box transcription factor 5 (*SOX5*) pathway and block the malignant development of CDDP resistant GC (55) (Figure 1; Supplementary Table S1).

### The 5-fluorouracil resistance of circRNAs in gastric cancer

The 5-fluorouracil (5-FU) was first synthesized in 1957 and quickly became a routine anticancer drug that remains essential in many chemotherapy regimens today (56). The 5-FU cytotoxicity occurs due to the mistaken entrance of fluoronucleotides into RNA and DNA as well as nucleotide synthetic enzyme thymidylate synthase (TS) inhibition (57). Furthermore, circRNAs may participate in these mechanisms to regulate the sensitivity of 5-FU in GC. Research has shown that autophagy dysregulation may be one of the main reasons for drug resistance in cancer, in which circCPM participates (31). CircCPM was overexpressed in 5-FU resistant GC cell lines and tissues. It improves the expression level of protein kinase AMP-activated catalytic subunit alpha 2 (*PRKAA2*) by directly binding to miR-21-3p and leading to the activation of autophagy and chemoresistance (31). In addition, silencing circCPM can improve chemosensitivity of 5-FU in GC (31). Emerging proof has shown that glycolysis plays an important role in hypoxia-induced chemoresistance. HIF-1α is one of the major regulators in the metabolic process. Downregulation of circNRIP1 can sensitize GC cells to 5-FU under hypoxic conditions (58). The miR-138-5p is a circNRIP1 sponge that can inhibit the effect of hypoxia-induced 5-FU resistance *via* circNRIP1 through HIF-1α (58) (Figure 1; Supplementary Table S1).

### Oxaliplatin resistance of circRNAs in gastric cancer

Oxaliplatin (OXA) is an alkylating agent that inhibits DNA replication by forming adducts between two adjacent guanine or guanine and adenine molecules and is the third-generation derivative of platinum complex anticancer drugs. At present, the resistance mechanism for GC to OXA is very complex, involving microenvironmental, cell mitochondrial, cell membrane, cell protein, signal transduction, and other factors related to tumor growth. Morevoer, circRNAs are a novel landmark in OXA resistance in GC (59). The Gao et al. study has led to the identification of circSLAMF that promoted GC oxaliplatin resistance by regulating the miR-502-5p/*ADAM9* (ADAM metallopeptidase domain 9) axis (60). CircSLAMF is overexpressed in OXA-resistant tissues and cells. In addition, miR-502-5p efficiently inhibited OXA resistance in OXA-resistant GC cells, reversing the promoting effect of circSLAMF (60). Finally, *ADAM9* upregulation may act as an oncogene aggravating OXA resistance to GC treatment (60). CircCEP128 is highly expressed in OXA-resistant GC cells and its exosomes. It has the ability to boost OXA resistance of GC cells partly *via* the miR-515-5p/*SOX9* (SRY-box transcription factor 9) pathway, which is a promising therapeutic target (61) (Figure 1; Supplementary Table S1).

### Paclitaxel resistance of circRNAs in gastric cancer

Paclitaxel (PTX) is a stable tubulin cytotoxic agent that has become the first-line treatment for various cancers, including GC (62). It can induce cellular processes by disrupting normal microtubule dynamics required for cell division and interphase processes and by inducing tumor necrosis factor-alpha gene expression, leading to apoptosis or programmed cell death (63). PTX resistance in tumors has been reported to be associated with increased drug efflux, alteration of intercellular signaling, tubulin mutation, and overexpression of β-tubulin isotype composition (64). In addition, circRNAs have been found to be associated with the mechanisms of PTX resistance. For example, circPVT1 downregulation can result in a noteworthy decrease in P-gp and GST-π, which can reduce cytotoxicity by reducing the concentration of antineoplastic drugs (65). As the target of circPVT1, miR-124-3p promotes sh-circ-PVT1-caused PTX sensitivity in PTX-resistant GC cells and the expression of zinc finger E-box binding homeobox 1 (*ZEB1*). Overexpression can promote PTX resistance of GC cells (65). Thus, circPLEC can interact with miR-198 in GC cells and through miR-198 to regulate the downstream gene mucin 19 and oligomeric (*MUC19*). MUC19 downregulation weakened GC resistance to PTX and improved the apoptosis of PTX-resistant GC cells (66). Therefore, circPLEC can promote PTX resistance through the miR-198/*MUC19* axis, which has the potential to be a therapeutic target (66) (Figure 1; Supplementary Table S1).

### Pemetrexed resistance of circRNAs in gastric cancer

Pemetrexed is an antifolate analog that inhibits enzyme thymidylate synthase (TS) and is also active on dihydrofolate reductase and glycine ribonucleotide folate-dependent enzymes, which forms an effective combination with OXA for locally advanced or metastatic disease treatment (67). Drug resistance is common in cancer. Thus, determining the mechanism of pemetrexed resistance is vital for improving treatment. Prior research has shown that cell drug resistance increased significantly in the circMTHFD2 overexpression group (68). In addition, miR-124 is a target of circMTHFD2 that can increase the protein expression of TS and ATP-binding cassette subfamily C member 11 (*ABCC11*) and decrease the protein expression of RCF1 (respiratory supercomplex factor 1, mitochondrial). There are also reports that *ABCC11* is a drug efflux pump used for drug metabolism. Thus, circMTHFD2 has the ability of enhancing the pemetrexed resistance in GC (68) (Figure 1; Supplementary Table S1).

### CircRNAs and gastric cancer targeted drug resistance

#### Trastuzumab

**FIGURE 3:**
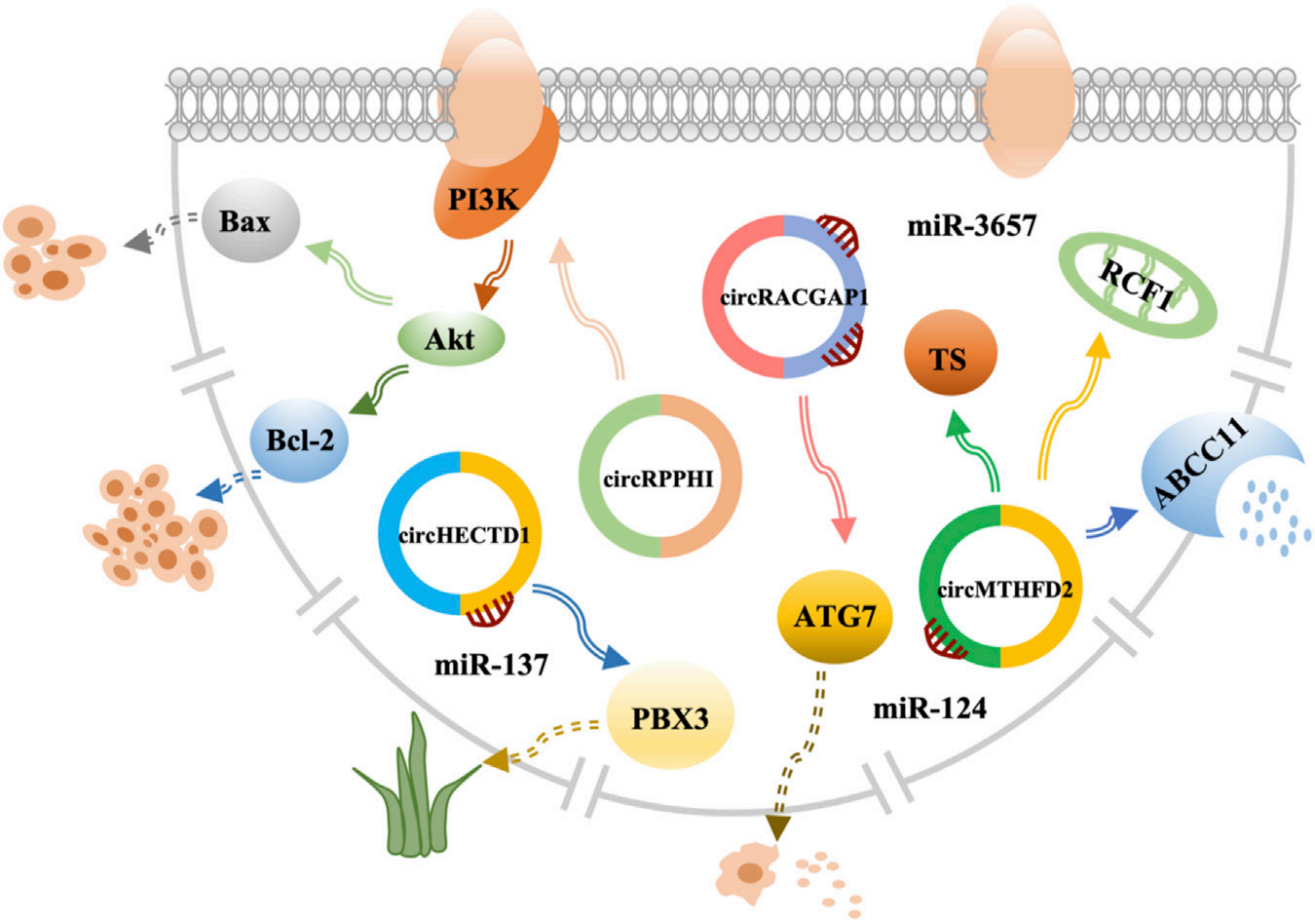
CircRNAs mediate the mechanism of drug resistance in GC.

Human epidermal growth factor receptor 2 (*HER2*) pertains to the epidermal growth factor receptor (*EGFR*), which is related to proliferation, apoptosis, and tumor cell migration (69). In addition, *HER2* is overexpressed or amplified in approximately 10%–20% of advanced GC cases (70). Therefore, trastuzumab is a new targeted drug that can be used in a combination of drugs to improve the treatment of advanced GC and has an important relationship with circRNAs. However, patients are prone to trastuzumab resistance. Therefore, research on the specific mechanism for the treatment of trastuzumab resistance has shown that circRPPH1 level was much lower upon trastuzumab treatment and had a low expression in cancer (71). Further research by Lv et al. has demonstrated that circRPPH1 had the ability to restrain proliferation and promote apoptosis in GC, indicating that it reduced trastuzumab resistance in GC cells (71). The study has also shown that cell viability of GC cells treated with signaling pathway activator insulin-like growth factor 1 (*IGF-1*) was significantly increased with a decrease in apoptosis (71). BCL2-associated X is an apoptosis gene that downregulates the expression, while anti-apoptosis gene BCL2 apoptosis regulator (*Bcl-2*) upregulates the expression (71). In conclusion, circRPPH1 can reduce trastuzumab resistance by inhibiting the phosphatidylinositol 3-kinase-threonine kinase 1 (*PI3K*)‐*AKT* in GC. Thus, it could be a new target for GC treatment (71) (Figure 3; Supplementary Table S1).

#### Apatinib

Apatinib is known as a highly selective inhibitor of vascular endothelial growth factor receptor-2 tyrosine kinase, which can induce apoptosis and inhibit proliferation and metastasis to participate in anti-angiogenesis and anticancer activities. It has been approved for treatment of metastatic GC (72). Increasing the efficacy of apatinib in GC has become a critical issue. Ling et al. have confirmed that apatinib promoted GC autophagy activation and downregulated the expression of autophagy-related gene 7 (*ATG7*) to sensitize apatinib in GC cells (73). Researchers have then selected circRACGAP1, which was differentially expressed in the apatinib and control groups, as the binding site for miR-3657 (73). Apatinib can increase *ATG7* expression *via* selective miR-3657 knockout. Using a co-transfecting plasmid and incubation with apatinib, the levels of autophagosomes and autolysosomes were reduced in circRACGAP1 loss GC cells and rescued *via* transfection with miR-3657 inhibitors (73). Therefore, the data indicated that circRACGAP1 regulated apatinib-induced autophagy by targeting miR-3657 and *ATG7*. Thus, circRACGAP1 can be used as a therapeutic target to regulate the role of apatinib in GC (73) (Figure 3; Supplementary Table S1).

#### Diosbulbin-bulbifera

Dioscorea bulbifera (DB) is a traditional Chinese medicine that is considered to be related to cancer treatment (74). Lu et al. have discovered that circHECTD1 is overexpressed in GC tissues and cells and serves as a sponge for miR-137 (75). Moreover, PBX homeobox 3 (*PBX3*) is a key cofactor that plays an important role in the carcinogenesis, development, and progression of GC (76), which is as downstream miR-137 gene target that is DB-resistant (75). These findings indicate that circHECTD1 prevents sensitivity *via* the miR-137/*PBX3* axis in GC (Figure 3; Supplementary Table S1).

## Conclusion and future perspective

Chemotherapy, targeted therapy, and immunotherapy are needed in advanced GC as they are able to provide some therapeutic effects (77). However, they inevitably result in drug resistance in the course of treatment, which will lead to a significant decline in treatment efficacy. Drug resistance has become one of the major obstacles to the therapeutic efficacy for GC. Therefore, it is crucial to explore all of the related mechanisms. As a new biomarker, circRNAs have been widely utilized in early screening of GC as they can be used as an miRNA sponge to regulate the downstream processes (78). Moreover, circRNAs play important roles in drug resistance through different mechanisms. In addition, circRNAs take part in the drug resistance process of target therapeutic drugs and even traditional Chinese medicines. Therefore, circRNAs have the potential to become new biomarkers of drug resistance in GC. However, their clinical application remains a challenge, as a large number of GC patient samples are necessary for detection and analysis. The method of measuring drug resistance of patients to GC by quantifying the expression of circRNAs also needs to be considered. At the same time, there are circRNAs that are abnormally expressed in multi-drug resistance, which makes identifying specific drugs a problem to be resolved. Therefore, a very important aspect of future research is to determine the specific molecular pathogenic pathways, which will provide more information about cancer and additional potential targets.
