# Supplementary material for: CircRNA: A new class of targets for gastric cancer drug resistance therapy
Source: Pathol Oncol Res. 2023 Mar 30;29:1611033. doi: 10.3389/pore.2023.1611033 (PMC10097900; doi:10.3389/pore.2023.1611033)
Supplement: Supplementary file 1 [file Table1.DOCX]

**Table 1 Circular RNA deregulation and roles in drug resistance of gastric cancer**

| CircRNAs | | Expression | | | Targets | | Function | | Drug | | Ref | |
| --- | --- | --- | --- | --- | --- | --- | --- | --- | --- | --- | --- | --- |
| Promoting drug resistance | | | | | | | | | | | | |
| circAKT3 | | | up | | miR-198/ PIK3R1 | | resistance | | Cisplatin | | [9] | |
| circCCDC66 | | | up | | miR-618/BCL2 | | resistance | | Cisplatin | | [37] | |
| circVAPA | | | up | | miR-125b-5p/STAT3 | | resistance | | Cisplatin | | [17] | |
| circFN1 | | | up | | miR-182-5p | | resistance | | Cisplatin | | [39] | |
| circASAP2 | | | up | | miR-330-3p/NT5E | | resistance | | Cisplatin | | [40] | |
| circPVT1 | | | up | | miR-30a-5p/YAP1 | | resistance | | Cisplatin | | [47] | |
| circPIP5K1 | | | up | | miR-299-3p/ENDOPDI | | resistance | | Cisplatin | | [48] | |
| circCOL1A2 | | | up | | miR-646/CDK6 | | resistance | | Cisplatin | | [49] | |
| circKRT7 | | | up | | miR-1200/POLD4 | | resistance | | Cisplatin | | [50] | |
| circDONSON | | | up | | miR-802/BMI1 | | resistance | | Cisplatin | | [42] | |
| circSMC3 | | | up | | miR-129-5p/MMP11 | | resistance | | Cisplatin | | [51] | |
| circFAM73A | | | up | | - | | resistance | | Cisplatin | | [52] | |
| circHNRNPU | | | up | | miR-637/CDX2 | | resistance | | Cisplatin | | [53] | |
| circRANGAP1 | | | up | | miR-449a/SHMT2 | | resistance | | Cisplatin | | [54] | |
| circLDLRAD3 | | | up | | miR-588/SOX5 | | resistance | | Cisplatin | | [55] | |
| circCPM | | | up | | miR-21-3/PRKAA2 | | resistance | | 5-Fu | | [31] | |
| circNRIP1 | | | up | | miR-138-5p/ HIF-1α | | resistance | | 5-Fu | | [55] | |
| circSLAMF6 | | | up | | miR-502-5p/ADAM9 | | resistance | | OXA | | [57] | |
| circCEP128 | | | up | | miR-515-5P/SOX9 | | resistance | | OXA | | [58] | |
| circPVT1 | | | up | | miR-124-3p/ ZEB1 | | resistance | | PTX | | [62] | |
| circPLEC | | | up | | miR-198/MUC19 | | resistance | | PTX | | [63] | |
| circMTHFD2 | | | up | | miR-124/MDR-1 | | resistance | | Pemetrex  ed | | [65] | |
| circHECTD1 | | | up | | miR-137/PBX3 | | resistance | | Diosbulbin-B | | [72] | |
| Inhibiting drug resistance | | | | | | | | | | | | |
| circCUL2 | | | down | | miR-142-3P/Rock2 | | sensitivity | | Cisplatin | | [36] | |
| circMCTP2 | | | down | | miR-99a-5p/MTMR3 | | sensitivity | | Cisplatin | | [38] | |
| circXPO1 | | | - | | miR-543/PHLPP2 | | sensitivity | | Cisplatin | | [41] | |
| circFAM114A2 | | | down | | ATM/Chk2/p53 | | sensitivity | | OXA | | [32] | |
| circRACGAP1 | | | up | | miR-3657/ATG7 | | sensitivity | | Apatinib | | [70] | |
| circRPPHI | | | down | | PI3K‐Akt | | sensitivity | | Trastuzumab | | [68] | |

5-Fu, 5-Fluorouracil; OXA, Oxaliplatin; PTX, Paclitaxel.
